# Proteome Biomarkers in Xylem Reveal Pierce’s Disease Tolerance in Grape

**DOI:** 10.4172/jpb.1000372

**Published:** 2015

**Authors:** Ramesh Katam, Kundai Chibanguza, Lekan M Latinwo, Danyel Smith

**Keywords:** Pierce’s disease, Proteome, Pathogenesis-related proteins, Xylem, *Vitis spp*

## Abstract

Pierce’s disease (PD) is a significant threat to grape cultivation and industry. The disease caused by bacterium *Xylella fastidiosa* clogs xylem vessels resulting in wilting of the plant. PD-tolerant grape genotypes are believed to produce certain novel components in xylem tissue that help them to combat invading pathogens. Research has been aimed at characterizing the uniquely expressed xylem proteins by PD-tolerant genotypes. The objectives were to i) compare and characterize *Vitis* xylem proteins differentially expressed in PD-tolerant and PD-susceptible cultivars and, ii) identify xylem proteins uniquely expressed in PD-tolerant genotypes. A high throughput two-dimensional gel electrophoresis of xylem proteins from three *Vitis* species identified more than 200 proteins with pls 3.0 to 9.0 and molecular weights of 20 to 75 kDa. The differentially expressed proteins were then excised and analyzed with MALDI/TOF mass spectrometer. The mass spectra were collected and protein identification was performed against the *Viridiplantae* database using Matrix Science algorithm. Proteins were mapped to the universal protein resource to study gene ontology. Comparative analysis of the xylem proteome of three species indicated the highest number of proteins in muscadine grape, followed by Florida hybrid bunch and bunch grape. These proteins were all associated with disease resistance, energy metabolism, protein processing and degradation, biosynthesis, stress related functions, cell wall biogenesis, signal transduction, and ROS detoxification. Furthermore, β-1, 3-glucanase, 10-deacetyl baccatin III-10-O-acetyl transferase-like, COP9, and aspartyl protease nepenthesin precursor proteins were found to be uniquely expressed in PD-tolerant muscadine grape, while they are absent in PD-susceptible bunch grape. Data suggests that muscadine and Florida hybrid bunch grapes express novel proteins in xylem to overcome pathogen attack while bunch grape lacks this capability, making them susceptible to PD.

## Introduction

*Vitis* (grapevines) is a genus of about 60 species of vining plants in the family Vitaceae, mostly is dominated by bunch (*Vitis vinifera* L., *V. labrusca* L., and other *Vitis* spp.) grape for commercial grape cultivation. Pierce’s disease (PD) is recognized as the most devastating grape disease caused by the bacterium *Xylella fastidiosa*, which is spread by xylem feeding leafhoppers known as “sharpshooters” [1]. PD is found abundance in southeastern US, where high temperature and humidity are common to the region.

While PD affects all *Vitis vinifera*-based cultivars, *V. rotundifolia* (muscadine) and other cultivars growing in the southern United States show tolerance to most various diseases, including the PD [2]. Muscadines are more popular for their nutraceutical value, because, they produce wide range of secondary metabolites [3]. Local PD-tolerant grape cultivars were hybridized with *viniferas* to develop new varieties of grape, known as Florida hybrid bunch (FH), which is also tolerant to PD [4]. But, their tolerance level varies compared to muscadine (whose tolerance remains stable) as the hybrids contain *V. vinifera,* a PD-susceptible species in their parentage. Although most commercial genotypes of grapevine are susceptible to PD, many wild *Vitis* genotypes and some hybrids of grapevine have shown strong PD resistance in greenhouse evaluations [5].

*Xylella fastidiosa* affects the xylem of grapevine by occlusion of the xylem vessels by biofilm formation, leading to water and nutrient stress and deterioration of the plant [6]. Xylem is important for the translocation of minerals and nutrients to various plant tissues. Xylem sap is known to contain various nutrients such as amino acids, sugars, organic acids, inorganic ions, proteins and low concentration of organic compounds which are essential to support bacterial growth [7]. Plants have responded to infection by altering the expression of certain classes of proteins that protect them from the pathogen [8]. Therefore, it is plausible to ascertain that these specific classes of proteins may be induced to protect the plants from pathogen invasion [9]. The presence of proteins in xylem and xylem sap has been reported in many plant species [10], and the number of proteins identified has increased considerably over the decade through the multi-parallel analysis of proteins [11,12]. Most cellular processes are regulated by protein-protein interactions, protein posttranslational modifications, and enzymatic activities, which cannot be identified by gene expression studies. Proteomics and bioinformatics tools are being applied increasingly as an approach to address biochemical and physiological inquires in response to biotic stresses in various plants [13–18].

Recent studies have shown that protein pattern of xylem sap changes in response to infection by pathogenic fungi, with some of the proteins being identified as pathogenesis-related [19]. No such studies have been reported for xylem sap in grape involving pathogen infestation. Xylem and xylem sap proteins of broccoli, rape, pumpkin, cucumber and tomato share homologies with several pathogen related (PR) proteins like glycine-rich proteins, peroxidase-like proteins, chitinase-like proteins, serine protease-like proteins, aspartyl proteases and lipid transfer-like proteins which are all active in the repair and defense reactions of the plant [20]. Comparative analysis of differential transcriptome associated with host-pathogen interactions from resistant and susceptible leaf, stem and shoot tissues of grape revealed the transcriptional pathways involved in host susceptibility and resistance in grape [21]. However, proteins and their pathways involved in host resistance in grape are not known. Several proteins such as peroxidases and chitinases have been found in xylem tissue of a variety of species suggesting a possible role in basic functions such as cell wall metabolism, lignification, cell death, and host-pathogen responses.

We have obtained a preliminary finding on number of differentially expressed proteins in xylem tissues using 2-DE [22], however, the identities of unique proteins were not investigated in the xylem tissue across the *Vitis* species. This study aims to better understand the nature and function of *Vitis* xylem proteins and their role in plant defense. The specific objectives of this research were to: 1) compare stem xylem proteome profiles of PD-tolerant and PD-susceptible grape genotypes, and 2) identify differentially expressed proteins playing a role in PD-tolerance among the PD tolerant and susceptible grape. Our study identified proteins unique and differentially expressed among three *Vitis* species and suggested their putative functions in PD-tolerance.

## Materials and Methods

### Plant material

PD-tolerant grapevines of Muscadine (*Vitis rotundifolia* cv. Carlos), Florida hybrid bunch (*Vitis vinifera* cv. Suwannee) and PD-susceptible *V. vinifera* (cv. Pinot Noir) were used in this study based on our previous proteomics analysis on *Vitis* leaf [23]. These cultivars were planted in the field with a 90 cms distance between each plant and a 150 cms distance between the rows of plants. Stem sections (25 cms) were collected from eight-week old grapevine shoots from six individual plants. Phloem was peeled off, and the xylem tissue was cut into 2.5 cms pieces, frozen with liquid nitrogen, and stored at −80°C.

### Total protein extraction

For total protein extraction of each sample, xylem tissues were collected from six individual plants and pooled together. Frozen xylem tissue was ground into a powder using liquid nitrogen and protein was extracted following the modified procedure [24]. Briefly, frozen powder (6 g) was vortexed in 20 ml of 50 mM Tris HCl (pH 7.5) containing 2 M thiourea, 7 M urea, 2% Triton X-100, 1% DTT and 4% PVPP. The suspension was centrifuged at 5000 rpm and the protein from the supernatant was precipitated with TCA (15%). Protein pellets were washed twice in cold acetone (−20°C) and centrifuged for 15 min at 13000 rpm. Final pellets were resuspended in IEF rehydration solution [7 M urea, 2% CHAPS (w/v), 2 M thiourea, 0.2% DTT (w/v)], and insoluble material was removed via centrifugation. The protein content of the extracts was determined according to the Bradford method [25].

### 2-DE Protein mapping

An aliquot (250 μg in 100 μl) of the protein extract was loaded on to the tube gels and isoelectric focusing (IEF) was performed as described previously [26]. Briefly, IEF tube gel was prepared using the ampholines (pH 3–10, 9–10.5; 5–7; 3–4.5; 2–4) supplied by BioRad. After the completion of IEF, the gels were equilibrated for 15 min in equilibration buffer [50 mM Tris-HCl, pH 8.8; 6 M urea; 30% (v/v) glycerol; 2 M thiourea; 2% (w/v) SDS; and 2% (w/v) DTT]. The equilibrated tube gels were then loaded onto a slab gel containing 12% (w/v) separating gel and 4% stacking gel (w/v). Electrophoresis was carried out in a BioRad Protein II slab system at a constant current of 20mA/gel. The gels were stained with colloidal Coomassie Brilliant Blue R-250 (0.25%) solution (50% methanol, 10% acetic acid and 40% distilled water) for 2 hrs. until the gel is uniform blue color. The gels were later, de-stained for 2–24hrs.in solution containing 5% methanol, 5% acetic acid, and 90% distilled water to visualize protein spots. The gels were stored in 7% acetic acid solution.

### Gel image and statistical analysis

Gels were scanned using Gel Image system (BioRad, Hercules, CA) and analyzed using PD Quest software, version 8.0.1 (BioRad) in order to detect significant, and consistency of the expressed proteins. For consistency, the gel area was defined using selected proteins bordering each side of the gels as landmark. In all cases this area corresponded to at least 95% of the total gel area. Spots across the gel replicates were matched by landmarks that label the spots present and positioned consistently, in all replicated gels. The Analysis Set, derived from three replicated gels of matched spots that were present on all the gels, was created, and the spots were analyzed and characterized.

Three independent replicates were performed per species and image analysis was carried out considering all gels. Initially, from each species, three replicated gels were analyzed by PD Quest (version 8.0.1) to normalize the gel images, and created a master gel profile. It should be noted that to derive a representative profile of each cultivar, three replicate gel profiles were obtained using their pooled xylem sample and then, each gel master profile was developed. The master profiles representing each of these species were compared to determine the differences in relative protein abundance among *Vitis* species studied. A one-way analysis of variance (ANOVA) was conducted to compare the mean protein spot densities and test if there was any difference in the protein spot abundance among the three *Vitis* species. The differentially expressed spots (with P-values <0.05) showing significant differences in the protein abundance time points were chosen for further analysis. Protein spots demonstrated a ratio of at least 1.5 fold between one another were defined as differentially expressed proteins [27]. Seventeen protein spots that showed significant differences in the spot densities and the spots showing at least 1.5 fold difference between one another were manually excised from gels and subjected to MALDI/TOF and database search for identification.

### In-Gel trypsin digestion

The digestion reaction included disulfide bond reduction with 10 mM DTT for 10 min at 60°C, alkylation with 100 mM iodoacetamide for 35 min at 25°C and digestion for 6.5 h at 37°C in 35 μl of 5ng/μl trypsin and 25 mM ammonium bicarbonate. The resulting peptide mix was desalted with C_18_ Zip Tips (Millipore), and 0.7 μl of the eluate [(peptides in the solution of 70% acetonitrile, 0.1% tri-fluoro-acetic acid and 5mg/ml matrix (α-cyano-4-hydroxycinnamic acid)] was spotted on the ABI 01-192-6-AB MALDI plate (Applied Biosystems, Foster City, CA).

### Mass spectrometry analysis

Mass spectra were collected on ABI 4700 Proteomics Analyzer (Applied Biosystems) MALDI/TOF mass spectrometer (MS) and protein identification was performed using the automated result dependent analysis of ABI GPS Explorer software, version 3.5 (Applied Biosystems).

### Database search, data analysis and protein identification

During the initial MS scan, data were analyzed as Peptide Mass Fingerprinting (PMF) and preliminary protein identifications (ID) were done by searching against the database using the MASCOT (Matrix Science) algorithm [28]. Furthermore, the GPS Explorer Software introduced unifying limit called Confidence Interval (C.I. %), which rates the confidence level of the MASCOT Protein Score or Ion Score (for each MS/MS event). The closer the Confidence Interval (C.I. %) is to 100%, the more likely it is that the protein is correctly identified. Proteins that were preliminarily identified by PMF with high confidence (confidence interval C.I.% > 95%) were subjected to *in silico* trypsin digestion, and their five most prevalent corresponding peptides–precursor ions present in the MS spectra were selected for MS/MS analysis known as RDA_1 (top protein confirmation). Both MS and MS/MS data were matched against the NCBI *Viridiplantae* taxonomic database. Only the proteins with a total score of C.I% > 95% were considered as positive ID.

### Gene ontology (GO) annotation

Mapping to UniProtKB: For functional analysis, the identified proteins were mapped to Universal Protein Resource (UniProtKB) to assess their functional analysis. The accessions were queried using batch Entrez to retrieve several sequences that mapped to different proteins. The annotations and accession numbers were retrieved using the GO Retriever tool and were grouped into different levels. Protein sequences were searched against gene ontology tools and TargetP program to derive functional classification and sub cellular localization [29].

## Results

### *Vitis* xylem proteome

Xylem is considered to be recalcitrant plant tissue for proteomic analysis due to its low protein content and the presence of interfering substances, which affects protein mobility, causing excessive streaking and preventing protein entry in to the IEF gel. Therefore, we have modified the protein extraction protocol by increasing the phenol separation time by 20min in ice-cold condition on rotary shaker. This modification yielded purified protein suitable for 2-dimensional electrophoresis (2-DE). Protein yield among *Vitis* species ranged between 2 to 2.5mg/g from the xylem tissues. The 2-DE resolved xylem proteins into more than 200 specific proteins with pIs between 5.0 to 9.0 and molecular weights (M_r_) ranging from 20 to 75kDa. The majority of the xylem proteins had a M_r_ between 30 and 75k Da and resolved into multiple spots. The three *Vitis* species showed major differences in proteins with M_r_ ranging between 20 and 75kDa and pIs between 5.0 and 8.0. The highest number of proteins was found in *Vitis rotundifolia* (muscadine), (ca. 245) followed by *Vitis vinifera* (Florida hybrid bunch), (ca. 215) and *Vitis vinifera* (bunch) (ca. 185).

### Differentially expressed xylem proteins among *Vitis* species

**Figure 1:**
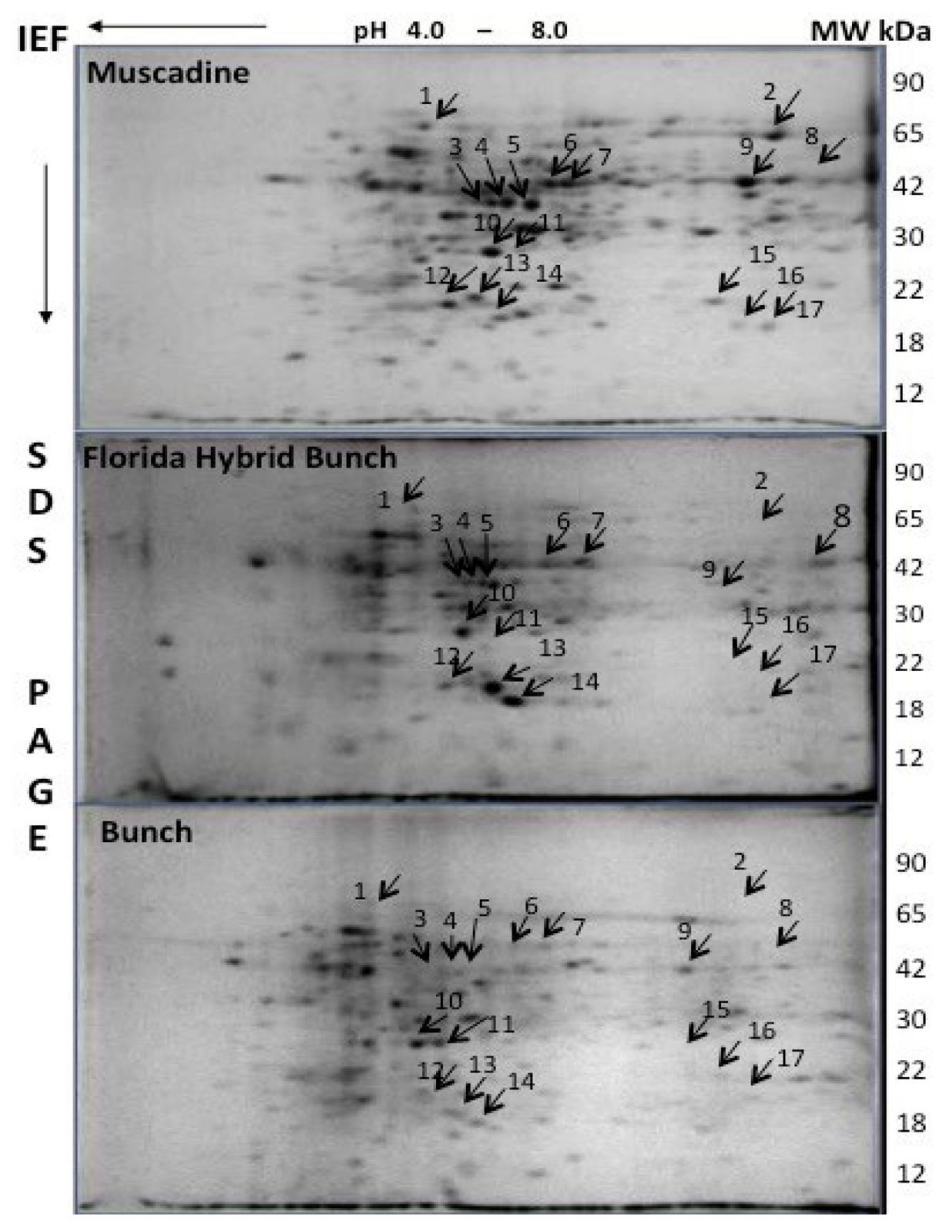
Differences in Xylem Protein Composition Among *Vitis* Species (Muscadine: cv. Carlos, Flroida Hybrid bunch: Suwannee, Bunch: cv. Pinot Noir). Proteins showing significant variation in composition are shown with arrows.

**Figure 2:**
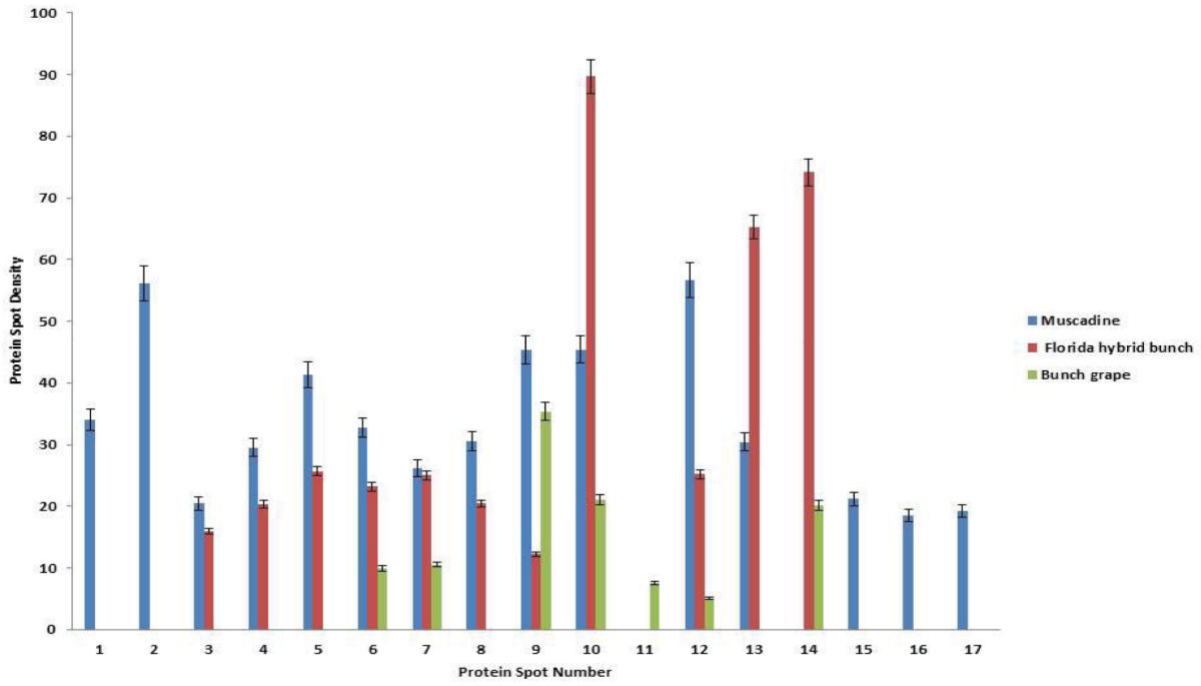
Quantitative differences among differentially expressed xylem proteins among *Vitis* species-*Vitis rotundifolia* (Muscadine cv. Carlos), *Vitis* spp. (Florida hybrid bunch cv. Suwannee), and *Vitis vinifera* (Bunch cv. Pinot Noir).

**Figure 3:**
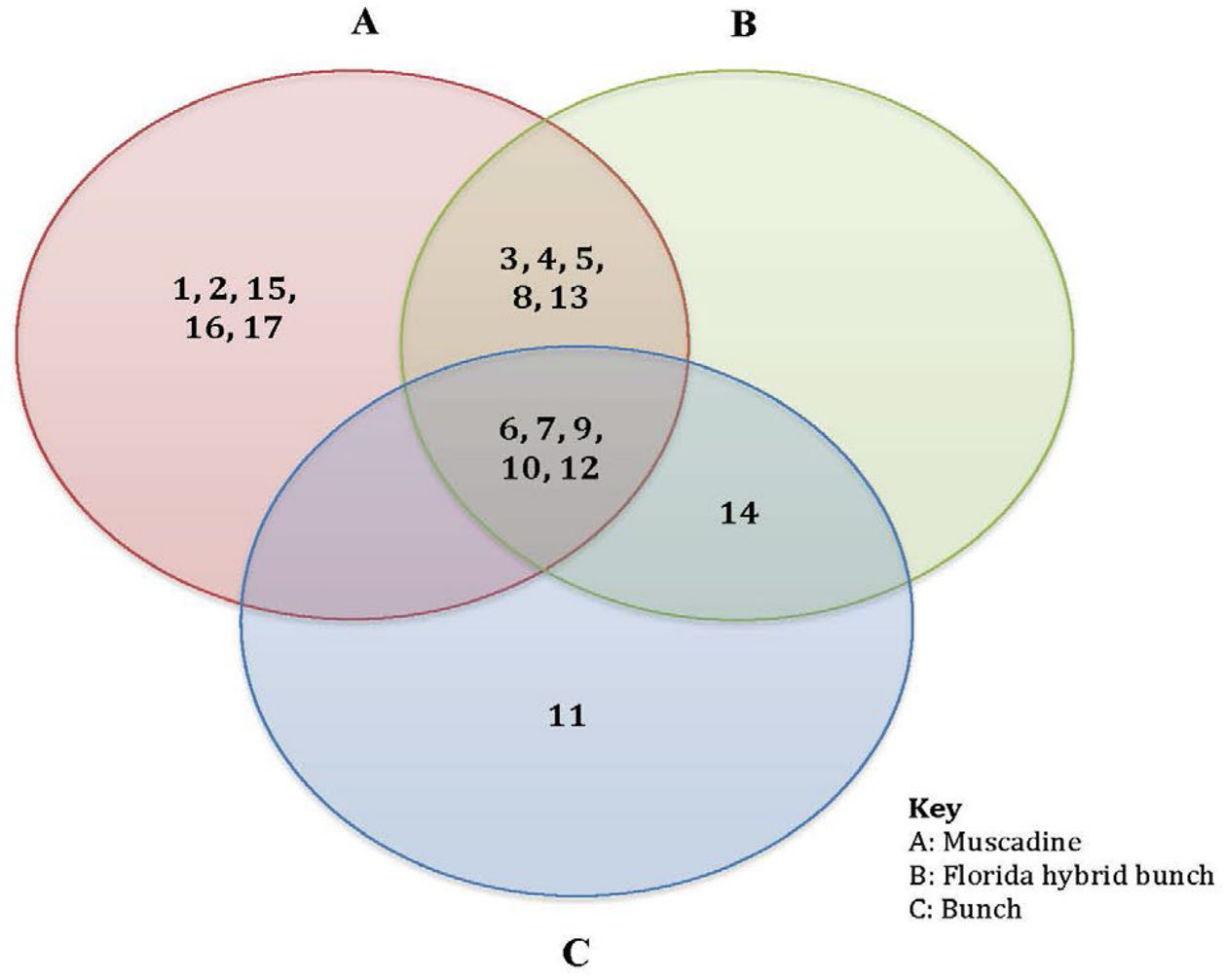
Venn diagram showing the distribution of differentially expressed proteins among *Vitis* species-*Vitis rotundifolia* (Muscadine cv. Carlos), *Vitis* spp. (Florida hybrid bunch cv. Suwannee), and *Vitis vinifera* (Bunch cv. Pinot Noir).

The comparative proteome analysis of the three species showed significant qualitative and quantitative differences among the *Vitis* species (Figure 1). Among 17 differentially expressed proteins, protein spot #10 was more abundant within Hybrid bunch, followed by muscadine and bunch grape. In addition, five proteins (#1, 2, 15, 16 and, 17) were present in muscadine cultivar but were absent in Florida hybrid bunch (FH) and bunch grape cultivars (Figure 2). The distribution of all 17 proteins among three cultivars is shown in Venn-Diagram (Figure 3).

### Identification and characterization of Vitis xylem proteins

The differentially expressed proteins were subjected to trypsin digestion followed by MALDI/TOF analysis to determine their identity. The peptide sequence tags generated for each spot from the MALDI/TOF analysis was used for protein identification through MASCOT sequence query search. The data were matched against the NCBI *Viridiplantae* taxonomic database. From here onward, the reference “protein identification” or “identity” denotes that MS spectra from this study matched to peptides belonging to a particular protein in the *Viridiplantae* taxonomic database (Supplement Table 1). Using this approach, 17 proteins that satisfied the selection criteria of 95% C.I. by MASCOT software were analyzed (see Materials and Methods) and listed in the Table 1. An extended BLAST search showed that proteins 12 proteins (# 3, 4, 5, 6, 8, 9, 10, 13, 14, 15, 16 and 17) showed homology to *Vitis vinifera.*

### Sub cellular localization and ontological classification of identified proteins

The identified proteins were grouped according to their cellular function. These categories include proteins associated with carbohydrate metabolic processes, disease resistance, energy metabolism, protein processing and degradation, biosynthesis, stress related functions, cell wall biogenesis, signal transduction and ROS detoxification.

## Discussion

Fewer proteomic studies have been carried out in grape xylem tissue, primarily due to technical challenges in extracting proteins from this plant matrix and due to the intrinsic complexity of most pathosystems [30]. The focus of this research was to identify major differences in xylem proteome among the selected three popular cultivars of *Vitis* species. The major functional group proteins showing quantitative and qualitative differences in xylem proteome include stress response, cellular biogenesis, signal transduction, energy metabolism and protein trafficking.

From this study, it is evident that muscadine and FH (known to be PD tolerant) cultivars have relatively more proteins and in high abundance compared to bunch grape. Out of 17 differentially expressed proteins, a total of 16 proteins were found in either muscadine or FH cultivar(s) or both. Muscadine and FH cultivars showed relatively more abundant proteins involved in carbohydrate and energy metabolism, secondary metabolism, protein processing and degradation, stress and ROS detoxification. Five proteins were found in all the three *Vitis* species, while twelve (12) proteins were absent in either one or two species.

### Proteins Unique to Muscadine Grape cultivar

Five proteins (Putative β-1, 3-glucanase, 10-deacetyl baccatin III-10-O-acetyl transferase-like, signalosome protein (COP9), aspartyl protease nepenthesin precursor are present only in muscadine grape and appear to be unique to this species cv. Carlos. These proteins are known to be involved in defense, stress, signal transduction and cellular biogenesis (Table 1). Putative β-1, 3-glucanases (spot #1 and 2) are widely distributed among higher plants and function as components of various specialized cell walls. Putative β-1, 3-glucanase proteins are deposited transiently at the cell plate during cell division, or are commonly associated with plasmodesmata and sieve plates. Callosic deposits, which are composed largely of β-1, 3-glucan are found between the plasma membrane and cell wall in infected or otherwise stressed plant tissues, and contribute to the formation of papillae that are associated with defense reactions in host-pathogen interactions [31]. In tobacco plants, inoculation with a pathogen had increased activity of PR proteins, [including β-1, 3-glucanase (PR-2)], which was associated with resistance to challenge inoculation with *P. tabacina* [32]. Similarly, pathogen infection or SA treatment induced extracellular secretion of β-1, 3-glucanases [33]. Therefore, it is plausible to ascertain that the presence of this protein in muscadine contributes to the increased resistance against the pathogens or abiotic stresses. 10-deacetyl baccatin III-10-O-acetyl transferase-like protein (spot #15) catalyzes the formation of last diterpene intermediate in taxol biosynthesis [34]. Accumulation of taxol, which is a secondary metabolite often occurs due to biotic or abiotic stresses such as elicitors or signal molecules play a major role in adaptation of plant in overcoming the stress condition [35]. The constitutively photomorphogenic signalosome protein (COP9-spot#16) is a component of the ubiquitin-proteasome system that regulates the activity of CULLIN-RING E3 ubiquitin ligases (CRLs). Several CRLs (or substrate receptors) have been assigned a role in signaling pathways, cell cycle, stress response, and pathogen defense [36]. CRLs ubiquitinate substrate proteins and thus target them for proteasomal degradation [37]. Aspartyl protease nepenthesin (NAP) (spot #17) has been characterized as an extracellular endopeptidase that mediates a peptide signal system in the activation of inducible resistance mechanisms in *Arabidopsis*. Although little information on this process is available so far, one of the NAP isoforms is the constitutive gene product known as cerebellar degeneration-related protein-1 (CDR1). CDR1 was hypothesized to mediate a peptide signal system involved in the activation of inducible resistance mechanisms in *Arabidopsis* [38].

### Proteins identified in PD tolerant Vitis species

We identified 4 proteins that are commonly found in two PD tolerant grape species. Proteins PR-10, subtilisin-like protease, 60 S acidic ribosomal protein P0, and heat shock cognate putative 70 kDa were identified in both muscadine and FH, but are absent in bunch grape (spot #3, 4, 5, 8, and 13). Majority of these proteins were in higher abundance in muscadine than in FH. Several defense-related proteins were induced by the pathogen, most of which were from the PR-10 (Spot # 3 and 4) proteins that are induced by biotic and abiotic stress in many plants. PR-10 proteins/transcripts were shown to increase abundantly in several biotic stresses in grapevine [39,40]. Plant subtilisin-like protease (subtilases) (spot #5) functions in the modification of plant morphology and the cleavage of cell wall structure proteins [41]. There is also evidence that subtilases in *Arabidopsis* are involved in responses to pathogens and salicylic acid [42]. 60 S acidic ribosomal protein P0 (spot #8) is the stalk protein that involve in protein translational processes. The consistent binding ability of the stalk protein in tolerant cultivars may contribute to maintaining high concentrations of translation factors around the ribosome, thus promoting translational efficiency [43]. Heat shock cognate 70 kDa protein (spot #13) may also be regarded as transcription factors, but their specific role in disease resistance has not yet been clearly defined [44]. These HSPs could be systemically induced in grape plants and that they may play an important role in resistance to PD. The three HSPs identified in grape stem were predicted differentially expressed in *Xf*-inoculated PD-resistant and-susceptible genotypes [30].

### Protein identified in FH and bunch grape

Acid phosphatase (spot # 14) was in high abundance in FH and bunch grape, but was not detected in the muscadine cultivar. Acid phosphatase could play a role in dephosphorylating the apoplastic proteins xylosidase and glucosidase. These two proteins are responsible for the degradation of xyloglucan oligosaccharides in cell walls [45]. Therefore, it is not surprising that putative β-1, 3-glucanase was not detected in both FH and bunch grape.

### Miscellaneous proteins differentially expressed among Vitis species

In addition to the unique proteins identified in muscadine and FH cultivars, five other differentially expressed proteins (# 6, 7, 9, 10, and 12) were detected in all three *Vitis* species. Most of these proteins were expressed in high abundance in muscadine, FH and bunch in decreasing order, indicating that these expression levels are positively correlated with the disease resistance of the cultivar.

α-L-arabinofuranosidase/β-D-xylosidase (#9), a key enzyme for the complete degradation of xylan is present in higher abundance in Muscadines. This protein is. β-D-xylosidases release D-xylose from various natural cell wall polysaccharides and oligosaccharides or synthetic substrates [46]. Thaumatin like proteins (TLP-spot#10) are defined as pathogenesis-related proteins and are homologous to osmotins [47]. TLPs are normally expressed at low levels in healthy plants, but rapidly accumulate to high levels in response to biotic or abiotic stress. Therefore, overexpression of TLP in muscadine and FH is a mode of defense mechanism to biotic stress, which makes these two species more tolerant than bunch grape. It has been reported that infection of rice by *Xanthomonas oryzae* pv. Oryzae leads to high expression of TLPs in rice [48]. Over expression of fructose bisphosphate aldolase (#6) was evident after β aminobutyric acid (BABA) pre-treatment. The abundance of this protein species in muscadines might help BABA-treated plants in producing more reducing power to meet the high-energy demands of pathogen-challenged cells [49]. Chitinase-III (spot #7) was differentially expressed in all three species. Treatment with either the bacterial pathogen *Pseudomonas syringae* pv syringae or the SAR activators had been shown to induce the specific accumulation of chitinase III [50]. Thus, the inducible type III chitinase likely plays a complementary role in the defense against pathogen invasion in conjunction with the constitutive type IV chitinase, both locally and systemically. While peroxidase (spot #11 and 12) was found in higher amounts in muscadine compared to other two species, the specific association of this protein to PD is still not fully understood [51,52].

### Multiple spot identities on 2DE gel

In this study, we found 3 proteins identified as two different spots on 2-DE gel that showed similar molecular weights, but differ in pI values, as a result of protein modification. The three proteins are; putative β-1-3-glucnnase (Spot #1 and #2), PR protein 10 (spot #3 and #4), peroxidase (spot #11 and #12) that showed similar identity of protein indicating the commonly occurring isoforms on 2-DE gel. The observed MW of these protein spots are higher than their theoretical MW, hence there is a possibility of post-translational modifications.

Although 2-DE cannot indicate whether those isoforms correspond to different forms of the same gene product, it will certainly provide an opportunity to detect full-length protein expression, and post translation modifications of the proteins. However, due to the lack of complete coverage of protein sequence/s using MALDI TOF, there has been little success to identify isoforms and post-translational modifications. Isoforms could arise from alternate splicing of the same gene transcript but yielding different mRNAs and hence, different proteins. The functional specificities of these isoforms seem to arise mostly from their distinct sub-cellular locations and specific interactions with other proteins [53]. Further studies combining western blotting and MS based protein identification could make such analyses possible.

## Conclusion

The PD tolerance level among *Vitis* species was found to correlate well with their xylem protein composition. Our results suggests that muscadine and Florida hybrid bunch grape genotypes express certain novel proteins while bunch grape lacks these proteins, making the latter, susceptible to PD. The differences observed in the relative amounts of various proteins among the three *Vitis* species suggested that these variations might contribute to grape plant’s unique tolerance characteristics to biotic and abiotic stresses.

Muscadine xylem tissue displayed the highest amount of pathogen-induced protein expression while Florida hybrid bunch xylem expressed a moderate amount, both in sharp contrast to bunch xylem, which exhibited the lowest amount of pathogen-induced protein expression. Proteins related to defense and signal transduction were more abundant in muscadine when compared to the other two *Vitis* species. Florida Hybrid bunch grape that showed some degree of tolerance have shown proteome profiles similar to those of muscadine. These often sharply contrasting levels of protein expression suggest that a higher number of proteins and the occurrence of certain novel proteins may contribute to grape species’ PD tolerance.

Although the differences in xylem proteome of these cultivars do not represent entire species, these studies certainly suggest a certain degree of variation in xylem proteome among the *Vitis* species, which might contribute to their unique traits. However, further studies are necessary to compare the xylem proteome within the species and evaluate the cultivar variations if any, and validate these variations using independent study of each cultivar within the species. Additional studies from a larger number of cultivated and wild *Vitis* species analyzed during different maturity stages coupled with an analysis of healthy and infected tissue will provide more details on the function of proteins in grape stem that determine plant development, disease tolerance, photosynthetic efficiency and the enological value of individual grape species. Thus, comparative proteomics has the potential to aid in the understanding of physiological and biochemical variations among the *Vitis* species genotypes.

## Figures and Tables

**Table 1:** Deferentially expressed protein in *Vitis* species.

Spot #	Accession #^a^	Peptide sequence	Th^b^ pI/Mol.Wt	Exp^c^ pI/Mol.Wt	MASCOT Scores	% Cover- age^d^	Peptides matched^e^	Location^f^	Protein homolog	Relative abundance^g^		
Carbohydrate metabolic process										Muscadine	Hybrid bunch	Bunch
1	115463555	IYNQNLI NHVGR	5.9/34706.5	5.86/67789	345	44	8	S	Putative β-1,3-glucanase	34	0	0
2	115463555	IYNQNLI NHVGR	5.9/34706.5	6.36/63264	324	48	12	S	Putative β-1,3-glucanase	56.15	0	0
9	115460876	AIGEVV STEAR	6.4/80921.5	6.35/53702	121	65	9	S	α-L-Arabinofuranosidase/β-D-xylosidase	45.32	12.25	35.4
	**Resistance**											
3	225431844	AAVLDADNLIP VRPQAIK	5.6/22000	5.95/35600	187	62	15	M	Pathogenesis-related protein 10	20.44	15.97	0
4	225431844	AAVLDADNLIP VRPQAIK	5.6/22000	5.55/35600	215	58	13	M	Pathogenesis-related protein 10	29.55	20.33	0
10	ABD64682	(R)CPDAYSYPKDDQ TSTFTCPAGTNYEVVF	8.37/23205	5.6/36006	603	78	19	-	Thaumatin-like protein	45.44	89.67	21
**Energy metabolism**												
6	108864048	(R)ATPEQVSDYTLK	6.1/41606.8	5.94/44751	243	43	23	-	Fructose-bisphosphate aldolase	32.75	23.2	9.9
14	115469166	FSDPQP DYSAFR	6.5/55150	5.8/32875	580	54	4	S	Acid phosphatase	0	74.15	20.21
**Cell wall biogenesis and degradation**												
7	125551525	VLVGVVASPEADR	5.9/32535.2	5.45/35352	432	57	17	M	Putative chitinase	26.15	25	10.55
**Protein processing and degradation**												
5	115488928	(R)IGYQKPSLIES (R)QIR	5.6/46274.2	5.58/45225	89	48	15	M	Subtilisin-like protease	41.35	25.68	0
8	115474653	GDSIVLMGK	5.4/34377.9	6.58/44080	151	62	13	-	60S acidic ribosomal protein P0	30.56	20.43	0
**Oxido-reductases**												
11	115474059	(R)GFSVID NAK (K)MGNISPLTGT QG QIR	5.8/32890.1	5.86/35497	197	61	18	S	Peroxidase	0	0	7.57
12	115474059	(R)GFSVID NAK (K)MGNISPLTGT QGQIR	5.8/32890.1	5.36/34497	197	61	18	S	Peroxidase	56.75	25.2	5.08
**Protein folding and assembly**												
13	115486793	NINPDEA	5.1/71185.3	5.36/31348	106	74	23	M	Heat shock cognate 70 kDa protein, putative	30.45	65.23	0
		VAYGAAVQAAILSGEGNEK										
**ROS detoxification**												
17	115451209	VVFDVA NSR	6.6/4577.4	7.52/48096	364	57	16	M	Aspartyl protease nepenthesin precursor	19.24	0	0
**Others/Unknown**												
15	115438572	MAAAAA VAAVAA AAAAAEPTVSK	5.0/46498.4	7.25/36783	101	54	18	S	10-deacetyl baccatin III-10-O-acetyl transferase-like	21.19	0	0
16	115451343	ALLPDK STVLDR	5.0/45327.2	7.37/36974	175	47	10	NA	COP9	18.5	0	0

**Notes:**

Spot numbers are given by PDQuest Software according to Figure 1

a Accession number in NCBI or grape EST translated query databases and organism assignment after BLAST homology searches.

b Theoretical pI/MW

c Observed molecular mass (Dalton) and pI value determined on the gel

d % coverage of peptides to homologous protein sequence

e Number of peptides identified from MALDI/TOF

f Localization was predicted by TargetP (http:www.cbs.dtu.dk/services/TargetP/)

g Unit of protein abundance determined using PDQuest

S: Contained a signal peptide in the secretory pathway

M: Contained a mitochondrial targeting peptide

-: Any other location

NA: Not applicable

FH: Florida Hybrid Bunch
